# Effectiveness of blended depression treatment for adults in specialised mental healthcare: study protocol for a randomised controlled trial

**DOI:** 10.1186/s12888-016-0818-5

**Published:** 2016-04-21

**Authors:** L. L. Kemmeren, D. J. F. van Schaik, H. Riper, A. M. Kleiboer, J. E. Bosmans, J. H. Smit

**Affiliations:** Department of Psychiatry, GGZ inGeest and VU University Medical Centre, P.O. Box 7057, Amsterdam, MB 1007 The Netherlands; EMGO+ Institute for Health Care and Research, VU University Medical Centre, Van der Boechorststraat 7, BT 1081 Amsterdam, The Netherlands; Department of Clinical Neuro- and Developmental Psychology, Faculty of Behavioural and Movement Sciences, Vrije Universiteit Amsterdam, Van der Boechorststraat 1, BT 1081 Amsterdam, The Netherlands; Department of Health Sciences, Faculty of Earth and Life Sciences, VU University Amsterdam, Van der Boechorststraat 1, BT 1081 Amsterdam, The Netherlands; Faculty of Health Sciences, The Institute of Clinical Research/Telepsychiatric Centre, Mental Health Services in the Region of Southern Denmark, University of Southern Denmark, Winsløwparken 19, DK-5000 Odense, Denmark

**Keywords:** Major depressive disorders, Internet-based intervention, Cognitive behavioural therapy, Blended treatment, Specialised mental healthcare, Routine practice, Randomised controlled trial, Cost-effectiveness

## Abstract

**Background:**

Internet-based interventions are seen as an important potential strategy to improve accessibility and affordability of high quality treatments in mental healthcare. A growing number of studies have demonstrated the clinical efficacy of internet-based treatment for mood disorders, but scientific evidence for the application in routine specialised mental healthcare settings is limited. Also, little is known about the clinical and health-economic benefits of blended treatment, where online interventions are integrated with face-to-face treatment of depression in one treatment protocol. The primary aim of this study is to investigate the clinical and cost-effectiveness of blended Cognitive Behavioural Therapy (bCBT) for depression, as compared to treatment as usual (TAU) in specialised routine mental healthcare in the Netherlands. This trial is part of the E-COMPARED project which has a broader perspective, focussing on primary and specialised care in eight European countries.

**Methods/Design:**

The study is a randomised controlled non-inferiority trial with two parallel conditions: bCBT and TAU. The blended treatment combines individual face-to-face CBT with CBT delivered through an Internet-based treatment platform (Moodbuster). This platform includes a mobile phone application, used for ecological momentary assessments, automated feedback and motivational messages. Weekly alternating face-to-face (10) and online (9) sessions will be delivered over a period of 19-20 weeks. TAU is defined as the routine care that subjects receive when they are diagnosed with depression in specialised mental healthcare. Adult patients ≥ 18 years old meeting DSM-IV diagnostic criteria for major depressive disorder will be recruited within participating outpatient specialised mental healthcare clinics in the Netherlands. Measurements will be taken at baseline and at 3, 6 and 12 months follow-up. The primary outcome will be depressive symptoms, measured with the PHQ-9 and QIDS. Secondary outcomes include health-related quality of life, mastery, treatment preference, working alliance, system usability, treatment satisfaction and possible negative side-effects. Moreover, a cost-effectiveness analysis will be conducted from a societal perspective and will include both direct and indirect healthcare costs.

**Discussion:**

The results of this study will provide insight into the health and economical outcomes of blended treatment for depression and give an indication of the value of implementing blended treatment in specialised clinical settings.

**Trial registration:**

Netherlands Trial Register NTR4962. Registered 05-01-2015.

## Background

Internet-based interventions are seen as an important potential strategy to improve accessibility to high quality treatments in mental healthcare. Especially for depression the need for greater access to cost-effective treatments is stressed out, given the increasing social and economic burden of this disorder [1]. Depression is highly prevalent and has a severe negative impact on wellbeing, quality of life and social and work-related functioning [2]. The World Health Organisation (WHO) has predicted that depression will be the foremost overall cause of disability in developed countries by 2030 [3]. Despite the presence of several efficacious psychological and pharmacological treatments, many depressed individuals remain untreated [4]. It is estimated that in the Netherlands 40 % of depressed individuals do not receive or seek adequate care [5]. Deployment of more internet-based interventions may help bridge this gap.

A growing number of studies have demonstrated the clinical efficacy and the potential cost-effectiveness of internet-based treatment for mood disorders in controlled research settings [6–9]. Furthermore, internet-based treatment with professional guidance has found to be more effective than unguided delivery [10, 11]. A recent meta-analysis even showed, based on a yet limited number of studies, that guided internet-delivered cognitive behavioural therapy (iCBT) results in similar overall effects as regular face-to-face treatment [12].

Only a few studies have directly compared Internet interventions with face-to-face interventions for depression. In an experimental setting, Wagner et al. [13] compared similar treatment modules based on cognitive behavioural therapy (CBT) in a guided online format with a regular face-to-face format. Another randomised controlled trial (RCT) compared group-based face-to-face CBT to guided iCBT [14]. In both non-inferiority studies no significant differences between the treatment modalities were found on treatment outcome, indicating that guided iCBT might be at least as effective as comparable face-to-face delivered interventions.

Most of the above mentioned evidence comes from efficacy trials conducted in controlled research settings. Whether these promising results can be transferred to routine clinical practice is less well known. Patient populations in routine practice are often more heterogeneous in terms of their characteristics, preferences and comorbidity levels than the populations and services in controlled research samples. In a review of Andersson and Hedman (2013), no RCT and only two uncontrolled open studies examining the effectiveness on guided iCBT for depression in routine practice were identified [15, 16], with medium-large within group effect sizes. Although available evidence suggests that iCBT may be as effective in routine practice as it is in randomised controlled clinical trials [17–20], there clearly is a need for more effectiveness studies on guided iCBT for depression in routine care. Moreover, most studies so far were conducted among self-referred depressed individuals from the general population or in primary care. It is not yet clear whether guided iCBT is a suitable intervention for the more complex patients that are treated in routine specialised mental healthcare settings.

The question is whether the format of guided iCBT should be adapted for the application in routine specialised mental health care. In former studies, therapist support in guided iCBT was usually delivered through e-mail, chat, telephone or video conference [21]. From a clinical perspective it seems rational that patients with more severe and more complex symptomatology of depression as present in routine care, may need face-to-face interaction [22]. Therefore, a so called ‘blended’ treatment might be more appropriate for these settings, where online and face-to-face sessions are integrated into one treatment protocol [23–25]. The face-to-face part of this treatment ensures that the patient benefits from a supportive therapeutic relationship and more social control, that is likely to increase motivation to adhere to and complete treatment [26]. Therapists can help tailor online treatment by meeting specific individual patient’s needs and wishes, have the opportunity to probe more deeply and to give immediate responses. Face-to-face interaction also involves non-verbal cues, which can be crucial in the communication between patient and therapist. The online part of blended treatment means that patients have 24/7 access to treatment modules, offering more flexibility. In addition, mobile applications can support the therapy by real-time monitoring of patient’s state (“ecological momentary assessment”) and by personalised feedback based on user data (“ecological momentary intervention”) [27–29]. Blending online and mobile components with face-to-face therapy could improve patients’ active participation in the treatment, increasing self-reliance and self-management competencies, which subsequently may contribute to better long term results [13]. By extending the reach of the therapy into the daily life of patients, the number of face-to-face sessions required could be reduced, resulting in a decrease of costs. Additionally, waiting times caused by limited availability of clinicians could be reduced when part of the face-to-face sessions are replaced by online treatment, which means therapists can treat more patients in a given time period. Also, other challenges patients meet when seeking traditional care can be tackled with online treatment, such as inconvenience of session times, travel time and -costs or mobility issues [30, 31].

Until now, only a few studies have investigated blended treatment formats [32–34]. Preliminary findings are promising, indicating that blended treatment can be effective in the reduction of depressive symptoms. However, to date, no data are available on the effectiveness of blended depression treatment compared to traditional face-to-face therapy in routine specialised mental healthcare. To investigate whether blended treatment for depression in routine (specialised) mental health care may be an attractive solution to overcome the aforementioned challenges, the European Commission has granted a large European study (the E-COMPARED project: ‘European Comparative Effectiveness Research on Internet-Based Depression Treatment’), in which eight European countries will conduct a similar RCT to assess the clinical and cost-effectiveness of blended treatment for depression compared to treatment as usual (TAU) [35]. The proposed study is part of the E-COMPARED project and focusses on the RCT in the Netherlands.

### Trial objectives

The overall objective of E-COMPARED is to provide mental health care stakeholders including policymakers, patients, health care professionals, health insurers and mental health service providers with evidence-based information and recommendations about the (cost-)effectiveness of blended treatment for depression. Within E-COMPARED data will be pooled across eight participating countries, with a total of 1200 participants. This large clinic based sample generates statistical power to analyse treatment impact in terms of depression outcomes and cost-effectiveness as well as in terms of moderators and mediators of outcome, thereby investigating what treatment works best for whom [35, 36].

This protocol describes the RCT in the Netherlands. The primary objective is to evaluate the clinical and cost-effectiveness of blended Cognitive Behavioural Therapy (bCBT) for adults with a diagnosis of Major Depressive Disorder (MDD), as compared to treatment as usual (TAU). We expect the blended treatment to be at least as effective as the regular treatment for depression in routine specialised mental healthcare (non-inferior), but that the blended form can be offered at lower costs than TAU. Furthermore, the study aims to explore which patients are likely to benefit from this particular kind of treatment delivery and how to tailor blended depression treatment to individual patients, related to their characteristics.

## Methods and design

### Study design

This study is a two-arm parallel non-inferiority randomised controlled trial, comparing internet-based bCBT to routine care that patients receive when treated for MDD in outpatient specialised mental healthcare (TAU). The protocol for this study has been approved by the Medical Ethics Committee of the VU University Medical Centre (registration number 2015.078).

### Inclusion and exclusion criteria

In order to be eligible, participants must be ≥ 18 years old, have a primary diagnosis of Major Depressive Disorder according to DSM-IV criteria as confirmed with the MINI International Neuropsychiatric Interview (M.I.N.I) [37], and have a score above 5 on the Patient Health Questionnaire (PHQ-9) [38]. Participants will be excluded if meeting any of the following criteria: 1) having acute risk of suicide, assessed clinically by trained interviewers with the M.I.N.I.; 2) having serious psychiatric co-morbidity (e.g. bipolar disorder, psychotic illness or substance dependence) that requires alternative treatment, primary to the treatment of MDD; 3) participating in other psychological treatment for depression, parallel to the intervention treatment of the study; 4) insufficient comprehension of the spoken and written Dutch language; 5) not having access to a computer or tablet with internet; 6) not willing to carry an Android smartphone during the treatment period (made available by the research team when they do not have an Android smartphone themselves); 6) not willing to be randomly assigned to one of the two treatment groups, bCBT or TAU.

For applicants that are at high risk for suicide, relevant information and telephone numbers are provided and therapists are notified such that standardised procedures within the participating mental healthcare centres can be followed. When stabilised, the patient can still potentially be eligible to participate.

### Recruitment

Participants will be recruited within participating specialised mental healthcare centres in the Netherlands. For specialist mental healthcare, patients need a referral from a general practitioner or primary mental healthcare professional. Referred patients first undergo an intake interview by mental healthcare specialists, who determine the diagnosis and treatment plan. During the intake procedure, clinicians will ask all new patients with a primary diagnosis of MDD if they are interested to participate in the study, and provide them with a letter containing detailed information about the study and an informed consent form. Patients who have agreed to be approached by the researchers, will be contacted within a week by telephone and after going through the study information asked whether they are willing to participate. If affirmative, the researcher first screens whether some major study requirements are fulfilled (sufficient command of the Dutch language; no other parallel psychotherapeutic treatment for depression; having access to the internet; willing to carry an Android smartphone during the treatment period; and willing to be allocated randomly to the treatment condition) and the patient will be asked to sign the informed consent form and return it by post. If the initial criteria are fulfilled, the M.I.N.I. diagnostic interview will be conducted by a trained interviewer by telephone to determine depression status and co-morbidity. When eligible, and after receiving the signed informed consent, the researcher sends an e-mail to the patient containing an invitation with a link to fill out the online questionnaires. After completion of the baseline assessment, the patient is randomised to either bCBT or TAU. Patients are informed about the randomisation outcome by the researchers via telephone and therapists via e-mail. Treatment starts as soon as possible after the inclusion, preferably within two weeks. Participants will be informed that they can withdraw from the study at any time, without any statement of reasons and without any consequences for their subsequent treatment.

Patients who are not eligible to participate will be notified and remitted to regular treatment trajectories within the participating mental healthcare centres.

### Randomisation and blinding

**Fig. 1:**
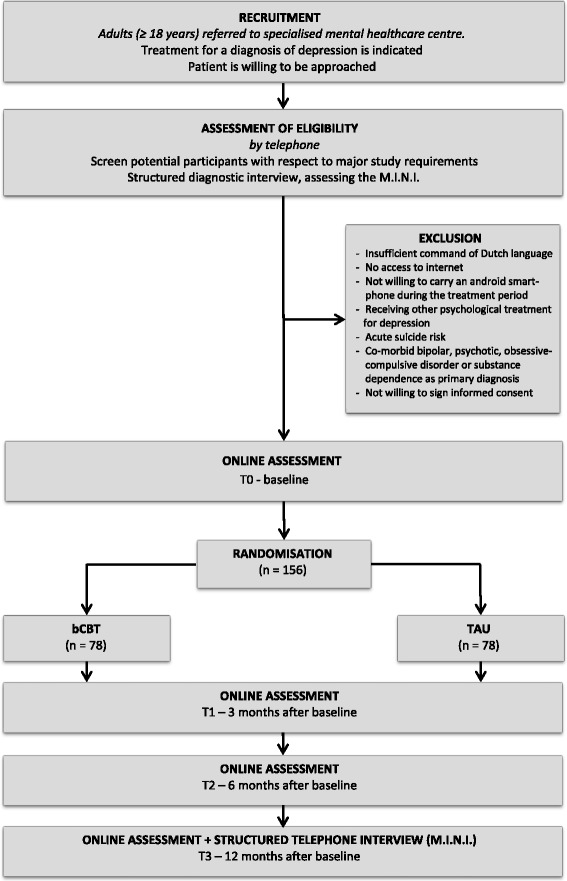
Study flowchart

Randomisation will take place at an individual level stratified by mental healthcare centre. The allocation will be conducted centrally at the VU Amsterdam by an independent team of researchers which is not involved in the trial. A computerised random number generator [39] is used to produce the allocation scheme with an allocation ratio 1:1. Subjects will be randomised into two groups: bCBT or TAU. The allocation is concealed, researchers and clinicians will be unknown to the randomisation scheme. Blinding for the treatment is not possible as it will be clear to both therapists and patients when the treatment is blended or not. However, the assessor conducting the M.I.N.I. at follow-up will be blinded to treatment status. The flowchart of the study is presented in Fig. 1, according to CONSORT guidelines [40].

### Blended intervention

The treatment manual is based on evidence-based Cognitive Behavioural Therapy (CBT) protocols implemented in routine practice [41] and recommendations in treatment guidelines for depression [42, 43]. The blended model provides CBT by integrating individual face-to-face (FTF) sessions and online sessions, supported by a smartphone application for the real-time monitoring of patients’ state in their natural environment (ecological momentary assessment: EMA). Through the mobile phone application, patients will also get automated motivational messages stimulating engagement on the platform as well as reminders to encourage treatment-related activities and improve compliance (ecological momentary intervention: EMI).

Patients receive ten FTF and nine online sessions over a period of 19–20 weeks, adhering to the usual time frame for the face-to-face CBT. The FTF and online sessions are weekly alternated (ratio 50/50). Communication between therapist and patient in-between the FTF sessions is asynchronous. The online part of the blended intervention is called “Moodbuster” [44].

### Online treatment platform

Moodbuster is based on the ICT4Depression platform (ICT4D), developed in a previous European FP7 project and applied in three small-scale clinical feasibility pilot trials [29, 45]. ICT4D was originally developed as a self-help system and is for the current project adapted to fit the blended format where therapist support is included. Previous to the proposed trial, a technical pilot of Moodbuster was conducted to ensure good system stability, responsiveness, functionality and usability in live trials. The ICT-platform Moodbuster is currently available in five languages: English, Dutch, German, Polish and French.

**Fig. 2:**
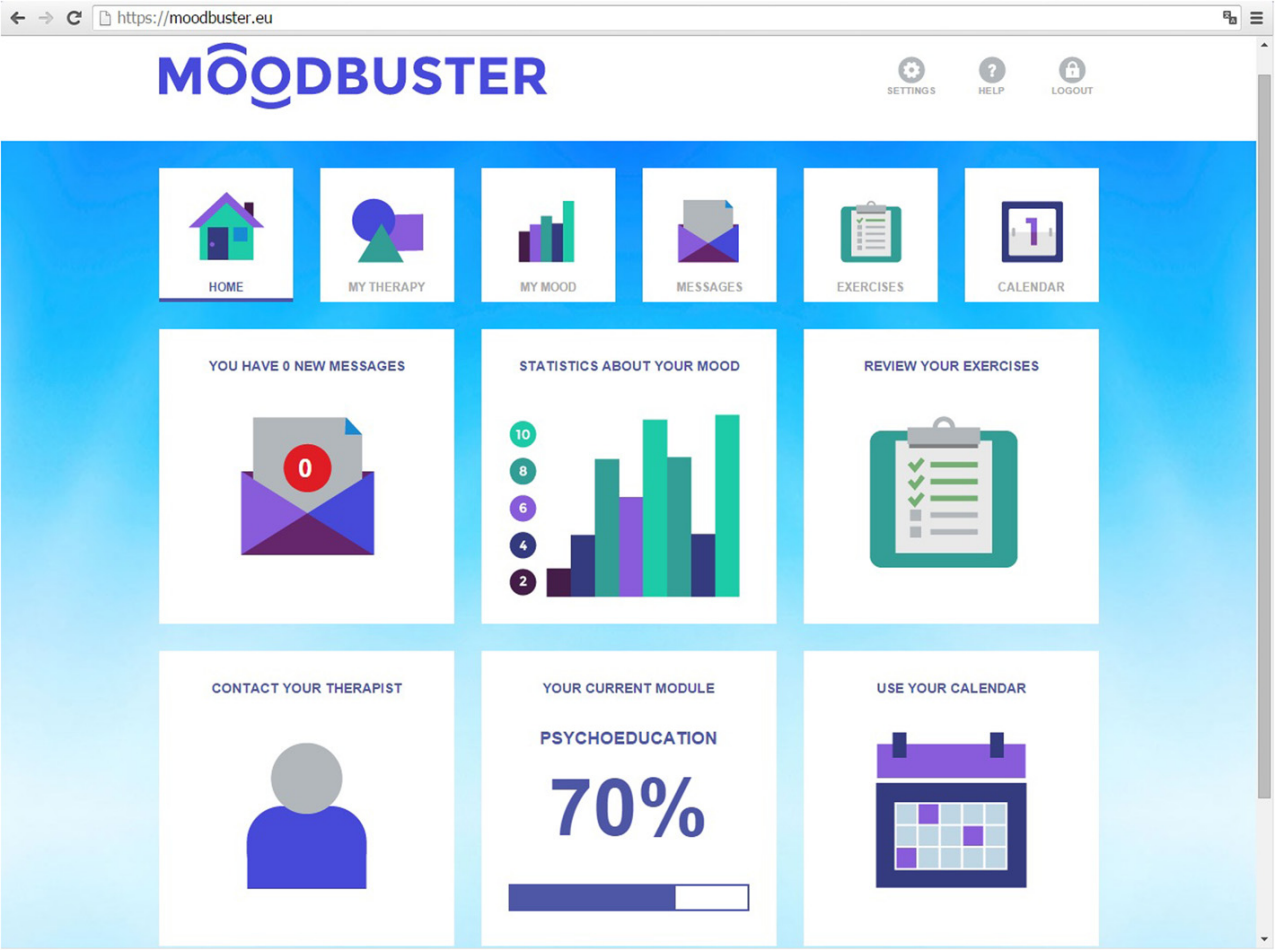
Moodbuster website: homepage of the patient portal

**Fig. 3:**
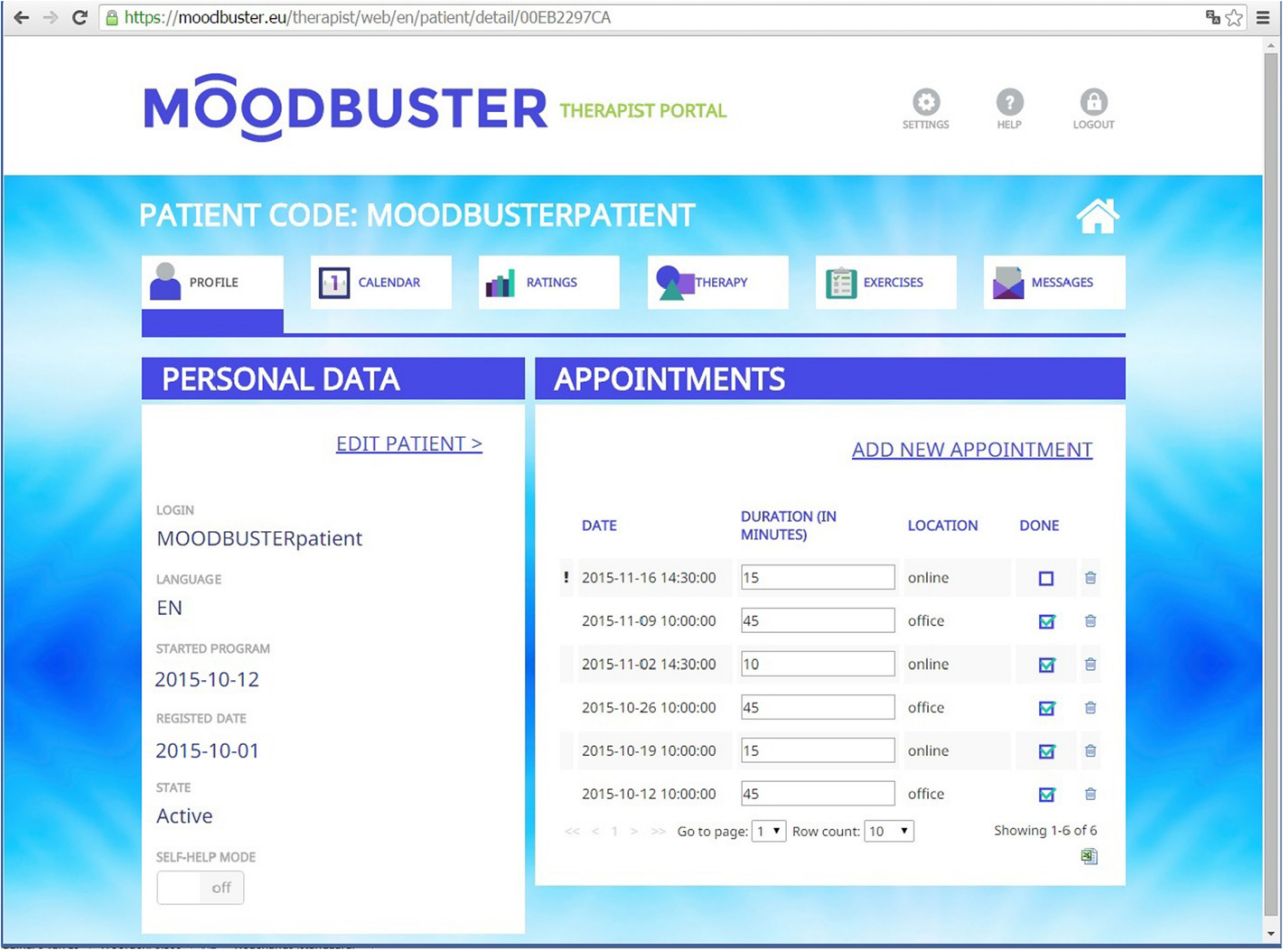
Moodbuster website: patient profile on therapist portal

The platform consists of 1) a *patient portal*: an online treatment environment for patients, with access to the treatment modules, homework exercises, mood graph, calendar and messaging system, 2) *a therapist portal*: a back-office for caregivers to monitor their patients and provide written feedback on exercises and progress in a secure way, and 3) a *mobile application* for real-time monitoring of mood state, cognitions, activities, social interaction, and sleep of the patient, as well as providing automated tailored reminders and motivational messages. Patients and therapists access the platform with a personalised log-in. Figures 2 and 3 show screenshots of the Moodbuster platform.

### Blended treatment protocol

Moodbuster contains an introduction module and six online treatment modules that are applied as integrated components of the FTF sessions within the blended treatment protocol. All treatment modules have the same structure, starting with an introduction and video about the content and purpose of the module, followed by didactical parts and exercises to apply the learned theory to own situations, and ending with a questionnaire assessing symptom severity and evaluation of the module. Patients work on one module at a time and get gradual access to the modules. The introductions of the modules are accessible at all time, but before entering a new module the patient has to confirm that the choice to activate that module was made in accordance with the therapist.

The blended treatment starts with the *Introduction* and *Psycho-education* module and ends with *Relapse prevention*. The order of the intermediate modules – *Cognitive restructuring, Behavioural activation*, *Problem solving skills* and *Physical exercise* – may vary, depending on the needs and preferences of the patient. The modules *Cognitive restructuring* and *Behavioural activation* are obligatory, as being part of the core components of the treatment. Therapists are required to include all four elements of CBT (psycho-education, cognitive restructuring, behavioural activation and relapse prevention), but are free to decide how many sessions are spent on each module. On completion of treatment, patients can continue to access Moodbuster to reread information and look up or repeat homework exercises.

Guidelines for the 45 min *FTF sessions* are to monitor and discuss depression symptoms based on mood ratings on the mobile phone, to review patients experiences with the homework exercises and the given feedback and to explain rationale and exercises within the current or upcoming module. The FTF sessions follow up on the CBT modules where content and exercises can be discussed in more depth, but also gives the therapist the opportunity to respond to the individual patient’s needs. Within the *online sessions* the patient works through a module and completes homework exercises. The therapist provides the patient with personalised written feedback on progress and content of exercises they have completed on the online treatment platform. The feedback is given at a scheduled time in-between the FTF sessions, via the Moodbuster messaging system. Providing online feedback will take therapists approximately 15 min. The *mobile phone application* will be used for EMA: the real-time monitoring of patients state in their natural environment. Patients will be prompted to rate their mood state, cognitions, activities, social interaction and sleep on a scale from 0–10 (from low to high). The EMA assessment questions and schedule are presented in Table 1. On the mobile application, patients will also receive automated motivational text messages to keep engaged with Moodbuster (rate mood and log in on platform), as well as reminders for scheduled activities within the modules and appointments with their therapist (EMI).

**Table 1:** EMA assessment questions and schedule

	Timing	Concept	Question
Full EMA diary	Morning diary	Sleep	How well did you sleep last night?
		Mood	How is your mood right now?
		Worrying	How much do you worry at the moment?
		Self-esteem	How do you feel about yourself right now
	Evening diary	Mood	How is your mood right now?
		Worrying	How much do you worry at the moment?
		Self-esteem	How do you feel about yourself right now
		Enjoy activities	How much did you enjoy activities today?
		Social contacts	How much were you involved in social interactions today?
		Pleasant activity level	To what extent did you accomplish pleasant activities today?
Mood rating	Random	Mood	How is your mood right now?

### Therapists

Either licenced CBT therapists or CBT therapists in training who work under supervision of an experienced CBT therapist will provide the blended depression treatment. All will receive extensive training on how to deliver the treatment (use of platform, skills for written feedback, blended protocol). To ensure treatment fidelity (1) a detailed treatment manual is available to guide therapists through the treatment, (2) regular supervision is provided between the therapists and the research coordinator and/or CBT supervisor and (3) therapists will fill in fidelity questionnaires after each session registering the (module) interventions used and the length of the contact. The number and frequency of sessions will be derived from the Electronic Patient Record Form and therapists’ activities on the platform will be assessed through track and change functionalities (log files).

### Treatment as usual

TAU is defined as the routine care that subjects receive when they are treated for depression in routine specialised outpatient mental healthcare. The type of treatment can vary and may consist of face-to-face psychotherapy (mainly CBT, interpersonal psychotherapy (IPT) or supportive therapy), antidepressant medication, running therapy, or a combination of these. We will not interfere with TAU but we will monitor carefully treatment utilisation through patient records, self-reported healthcare utilisation and fidelity checklist from therapists (see assessments). In both arms patients may receive medication; in this sense usual care is being followed.

### Assessments

Measures will be taken at baseline and at 3, 6 and 12 months after baseline. The diagnostic interview (M.I.N.I.) at baseline will be conducted by telephone by trained interviewers. At 12 months follow-up the depression, anxiety and PTSD sections of the M.I.N.I. will be assessed again by telephone by an interviewer blinded to treatment allocation. All other assessments will be performed online via a link to the self-report questionnaires sent by e-mail. Table 2 provides an overview of the measures and their time of assessment.

**Table 2:** Overview of measures

Variable	Instrument	T0^*^	T1^*^	T2^*^	T3^*^
*Measures taken from patient*					
Demographics (patient characteristics)		X			
Current and history of treatment		X			
Diagnosis of depression and comorbid disorders	M.I.N.I. 5.0	X			X
Depressive symptoms	PHQ-9	X	X	X	X
Depressive symptoms & severity	QIDS-SR-16	X	X	X	X
Health related well-being (QALY)	EQ-5D-5L	X	X	X	X
Health care uptake and productivity losses TIC-P	TIC-P	X	X	X	X
Sense of mastery	SOMS	X	X	X	X
Treatment preference		X			
Patient expectancy	CEQ	X			
Therapeutic alliance	WAI-SF-PT		X		
Technology alliance	WAI-Online Therapy^a^		X		
Client satisfaction with treatment	CSQ-8		X		
Satisfaction with the online program SUS	SUS^a^		X		
Possible negative side-effects	INEP			X	
*Measures taken from therapist*					
Therapeutic alliance	WAI-SF-TH^b^		X		
Satisfaction with the online program	SUS^c^		X		

^a^Offered to condition receiving blended treatment only

^b^Has to be completed for each patient. Offered at the same time point as T1 assessment of the patient

^c^Has to be completed once per therapist after completion of the first blended treatment

*T0 refers to baseline and T1, T2 and T3 to the 3, 6 and 12 month questionnaires

### Outcome measures

#### Primary outcome measure

The primary outcome measure is *symptoms of depression* as assessed with the Patient Health Questionnaire-9 (PHQ-9) [46]. The PHQ-9 is a nine-item mood module that can be used to screen and to diagnose patients with depressive disorders. The 9 items are each scored on a 0–3 scale, with the total score ranging from 0–27 and higher scores indicating more severe depression. The PHQ-9 has shown to have good psychometric properties [47, 48].

The Quick Inventory of Depressive Symptomatology Self-Report (QIDS-16-SR) is used in addition to the PHQ-9, because this measure is more often used (especially in specialised mental healthcare) and therefore makes better comparison with other studies possible. The QIDS assesses *depression severity* and consists of 16 items (each item scores 0–3, total range 0–48), with higher total scores being indicative of a higher severity of depressive symptoms. The QIDS includes symptom domains of MDD based on DSM-IV and Research Diagnostic Criteria (RDC). The QIDS has shown highly acceptable psychometric properties, with internal consistency of α = .86 [49]. The cut-off points of 6, 11, 16 and 21 represent the thresholds for mild, moderate, severe and very severe depression, respectively.

#### Secondary outcome measures

*A diagnosis of depression* will be assessed with section A and B of the *MINI International Neuropsychiatric Interview* (M.I.N.I.) version 5.0, a structured diagnostic interview based on the Diagnostic and Statistical Manual of Mental Disorders (DSM-IV) and on International Classification of Diseases (ICD-10) criteria [37]. In comparison with the patient-rated version of the SCID, the M.I.N.I. achieved a good Kappa score of 0.84 with a sensitivity of 0.96 and a specificity of 0.88 in diagnosing Major Depressive Disorder (MDD) [50].

The full M.I.N.I., with exception of section M (Anorexia Nervosa), N (Bulimia nervosa), and P (Antisocial personality disorder), will be conducted at baseline (T0) to determine lifetime and current depression, as well as current comorbid psychiatric. At 12 month follow-up (T3), the depression, anxiety and PTSD sections of the M.I.N.I. will be assessed again, covering the past 12 months.

*Demographic variables* including age, sex, ethnicity, marital status, educational level and employment status, as well as *history of treatment* for mental health problems will be assessed at baseline. In addition, patients will be asked about *treatment preference*, indicating which treatment alternative they prefer (bCBT or TAU) before being allocated to one of the two conditions.

*Patients’ expectancy of treatment* will be assessed after randomisation with the Credibility and Expectancy Questionnaire (CEQ). This questionnaire consists of six items measuring expectancies toward the therapy and the credibility of treatment options. The items are scored on one of two scales, one 9-point Likert scale ranging from 0 (not at all) to 9 (absolutely) or a continuous scale ranging from 0 % (not at all) to 100 % (absolutely). The sum score on credibility and expectancy can vary between 3 and 27, with higher scores being indicative of more positive attitudes. Both factors (credibility and expectancy) have shown to be stable across different populations, with high internal consistency within each factor [51].

*Locus of control* will be assessed with the 5-item version of the Sense of Mastery Scale [52], with proven good reliability [53]. Questions are scored on a Likert-scale ranging from 1 (‘totally disagree’) to 5 (‘totally agree’). The total score ranges from 5 to 25, with higher scores being indicative of a higher degree of perceived control. The Sense of Mastery Scale will be administered at all time points (T0-T3) to assess changes in locus of control.

*Patient’s satisfaction with the treatment* will be assessed with Client Satisfaction Questionnaire (CSQ-8), used to measure global patient satisfaction [54]. The questionnaire consists of 8 items that are measures on a 4-points scale. Total scores range from 8 to 32, with higher scores being indicative of higher levels of satisfaction. The CSQ-8 has high internal consistency of α = .93 [55].

*Satisfaction with the online platform* will be evaluated with the System Usability Scale (SUS), a simple 10-item questionnaire giving a global view of subjective assessments of usability of a technology system [56]. All items are measured on a 5-point Likert scale ranging from ‘strongly disagree’ to ‘strongly agree’. Total SUS scores have a range from 0–100. The questionnaire was found to be reliable and robust [57]. The SUS has to be completed by patients receiving bCBT only, and once per therapist three months after starting with their first blended treatment.

The *therapeutic alliance* between therapists and patient will be assessed with the short version of the Working Alliance Inventory (WAI-SF) [58], Dutch version [59]. The WAI-SF is a 12-item self-report questionnaire with responses on a 5-point Likert scale ranging from’never or rarely’ to ‘very often’. The questionnaire covers three dimensions of working alliance: 1) therapeutic goals, 2) tasks, and 3) bond. The subscales have shown to have good internal consistencies (α > .80). Both the patient and the therapist version of the questionnaire will be administered at T1, to determine the quality of the therapeutic alliance.

The *alliance between the patient and technologies* will be assessed with an adapted version of the WAI: the Working Alliance Inventory – Online Therapy, developed by Labpsitec (Labority of Psychology and Technology). The items address goals and tasks agreement with the online treatment program, and not with the therapist.

To assess possible *negative side-effects of the treatment*, the Inventory of Negative Effects of Psychotherapy (INEP) [60] will be administered at T2 (6 months after baseline). The version used in this study consists of 15 items, assessing a range of common changes participants experienced in line with their therapy, concerning their social and work environment. The following domains are covered: negative interpersonal changes, negative effects in an intimate relationship, family/friends, perceived dependence from therapist, stigmatisation. All items are rated on a 4-point Likert scale. For each item, respondents also state whether they attribute the adverse effects on the therapy or on other circumstances. Only item scores of those negative effects that were attributed on the therapy are added to the total score. Higher total scores indicate more negative effects. The INEP shows an internal consistency of α = 0.85 [60]. For the use in this study, the questionnaire was translated by the forwards-backwards method, i.e. the questionnaire was first translated from English into Dutch by two persons who reached consensus by discussion. Next, the questionnaire was translated back to English and was compared with the original questionnaire.

#### Economic evaluation

*Quality of life* will be determined with the five-level version of the EuroQol (EQ-5D-5L), a self-report questionnaire assessing the health related well-being of participants [61]. The questionnaire consists of five items: mobility, self-care, daily activities, discomfort and mood state. Each item has five response categories ranging from ‘no problems’ to ‘a lot of problems’ [62]. Furthermore, this scale contains a visual analogue scale concerning health state. The EQ-5D-5L health states will be converted to utility scores using the Dutch tariff. Quality-adjusted life-years (QALYs) will be calculated by multiplying the utility of a specific health state by the time spent in that health state. Changes in health states between measurements will be linearly interpolated. The EQ-5D-5L holds acceptable levels of content validity [63] and has demonstrated valid redistribution, reduced ceiling, and improved discriminatory power and convergent validity compared to the EQ-5D-3L [64].

*Health care utilisation* and *productivity losses due to illness* will be measured using an adapted version of the Trimbos and iMTA Questionnaires on Costs Associated with Psychiatric Illness (TiC-P), a self-report questionnaire consisting of two different parts that can be administrated separately [65]. Part I will be used to assess the participants’ healthcare utilisation and medication use. Part II (short form Health and Labor Questionnaire; SF-HLQ) consists of 11 items measuring lost productivity costs resulting from absenteeism (being absent from work because of illness) and presenteeism (being present at work while ill which may lead to reduced efficiency).

#### Engagement and usage measures for bCBT

Activities on the platform such as number of visits, time in-between logins and number of messages exchanged between patient and therapist, will be assessed through track and change functionalities (log files). Data on how patients use online modules (frequency, duration, order, completion), how they rate them and to what extent they adhere, will also be obtained through usage statistics on the Moodbuster online treatment platform.

Patients receiving the blended treatment will as part of the treatment protocol additionally fill out the PHQ-8 at the end of each module, for monitoring treatment outcome (i.e., all items on the PHQ-9 scale except the ninth item). As this data is being gathered in a self-administered fashion and direct action on positive responses on the ninth item inquiring “thoughts that you would be better off dead or of hurting yourself in some way” is not feasible, it was chosen to exclude this item. Patients are instructed to use existing services of the mental health care organisations when they feel the need to contact a doctor or a therapist outside working hours. The PHQ-8 has similar operating characteristics as the PHQ-9 (sensitivity, specificity, and positive predictive value), regardless of the threshold [38].

### Sample size

For the European study as a whole, the sample size calculation is based on the non-inferiority design and calculated for the primary clinical outcome symptoms of depression. The non-inferiority margin was set at ∆Cohen’s d = 0.20, which is a conservative estimate of the subjective minimal important difference that is noticeable by patients [66]. To determine that there is no difference between blended depression treatment and TAU on the primary clinical outcome, a total of 1052 patients is required to be 90 % certain (power of .90) that the lower limit of the two sided 95 % confidence interval will be above the non-inferiority limit of Cohen’s *d* = -0.2. To allow for expected drop-out and variation between settings, the total number of participants to be recruited will be 1200.

For the current trial in the Netherlands we aim to include 156 participants (78 per arm). This sample size will be sufficient to be 80 % sure (i.e. power = 1-ß = 0.80) that the lower limit of a one-sided 95 % confidence interval (or equivalently a 90 % two-sided confidence interval) will be above a non-inferiority limit of -0.4 if we assume that there is no difference between the standard and blended depression treatment. A margin of 0.4 was judged acceptable, as this range of small to moderate difference in effect size will not result in clinically important differences [67].

### Planned statistical analyses

In a non-inferiority trial, the study objective should be achieved in both the intention to treat and per-protocol population [68]. Analysis will be based on intention to treat (ITT) design, including all participants randomised in the study. Participants will be encouraged to provide assessment data, regardless of treatment adherence. Additionally, per-protocol (PP) analysis will be conducted, including only participants who followed the treatment protocol of the assigned treatment. Non-inferiority should be demonstrated also in the PP analysis, because ITT tends to dilute differences [69]. All analyses will be performed using the Statistical Package for the Social Sciences (SPSS), version 20.0.

#### Economic evaluation

We will perform an economic evaluation from societal perspective, conducting a cost-effectiveness analysis (CEA) and cost-utility analysis (CUA). The CEA will be based on treatment response, defined as a 50 % pre-post reduction of QIDS-SR depressive symptoms [49]. The CUA will be conducted using quality adjusted life years (QALYs) as a generic measure of health gains. Multiple imputation using chained equations (MICE) will be used to impute missing cost and effect data. Predictive Mean Matching will be used to account for the skewed distribution of costs [70]. Variables that will be included in the multiple imputation model are variables related to missingness, variables related to costs and effects at 12 months of follow-up and all variables included in the analysis model. The number of imputations will be increased until the fraction of missing information (FMI) is 5 % or less [70]. The completed datasets will be analysed separately and pooled using Rubin’s rules [71].

Multilevel analyses with adjustment for mental health care institution will be done to estimate differences in costs and effects between bCBT and TAU while correcting for potential effect modifiers and confounders. Incremental cost-effectiveness ratios (ICERs) will be calculated by dividing the difference in costs by the difference in effects. Bias-corrected and accelerated bootstrapping with 5000 replications will be used to estimate statistical uncertainty. Bootstrapped cost-effect pairs will be plotted on cost-effectiveness planes (CE planes) to visualize the uncertainty surrounding the ICERs. Moreover, for decision-making purposes cost-effectiveness acceptability curves will be estimated showing the probability that bCBT is cost-effective in comparison with TAU at a range of different ceiling ratios. Finally, to test the robustness of our findings, sensitivity analyses will be performed. First, the economic evaluation will be conducted from a healthcare perspective. Secondly, a per protocol analysis will be done in which only participants completing the bCBT treatment protocol will be included.

#### Clinical evaluation

Independent *t*-tests and *χ*2-tests will be used to estimate between-group differences in demographics and pre-treatment measures at baseline. Outcomes on continuous outcome variables at T1, T2 and T3 will be analysed via mixed-model analyses, with participants as random effects, and time (T1-T3), group (bCBT vs. TAU) and time x group as fixed effects, with baseline scores as a single covariate. To assess the magnitude of treatment effects, Cohen’s *d* effect sizes [72] for each time point will be calculated by dividing MM parameter estimates of fixed effects at each post-treatment assessment by the pooled standard deviation of baseline scores. Effect sizes under 0.2 are deemed to be small, 0.5 to be moderate and 0.8 to be large [72].

Differences in response and remission rates, as well as adherence rates will be examined using *t*-tests and *χ*2-tests. In addition, moderator analysis will be conducted for demographic variables such as gender, age, educational level, partner status, employment status, as well as treatment and patient characteristics.

## Discussion

The aim of this study is to evaluate the clinical and cost-effectiveness of blended CBT where face-to-face treatment is combined with internet and mobile technologies, as compared to treatment as usual for adults with MDD in routine specialised mental healthcare. Keeping depression treatment accessible, feasible and affordable is of high importance, considering the large individual and economic burden of this disease [1, 2]. The study described in this paper is the trial conducted in the Netherlands as part of E-COMPARED. The results of this study will provide insight into the health-economical outcomes of blended treatment and give an indication in the value of implementing blended treatment into routine specialised mental healthcare.

Although previous findings have shown similar effect of guided iCBT compared to traditional FTF treatment [17, 19], the current study will give an answer to the question whether this also holds for bCBT in more complex and severely depressed patients in specialised care, as well as whether bCBT is more cost-effective than regular depression treatments. Given that the number of required FTF sessions is reduced by replacing them with online sessions, it is assumed that bCBT might lead to cost savings. At the same time, waiting lists could be diminished as therapists can treat more patients in a given time period. The quicker treatment can start, the sooner people suffering from depression do recover and the sooner they are able to participate in society. Down the road, this could have a positive impact on the social economic consequences of depression seen that productivity losses due to the illness (absenteeism and presenteeism) is often the biggest expense [73, 74].

A major strength of the present study, is that it is one of the first to assess blended treatment instead of internet-based interventions used as standalone or add-ons, in the treatment of depression in routine practice. The assumption underlying the integrated approach within blended treatment, is that the benefits of online treatment are combined with the advantages of FTF therapy. However, many questions regarding a blended treatment format are still unanswered, e.g. on how to best blend the two modes of delivery, on program structure and flexibility or on frequency and content of therapist support [24, 25]. This study will contribute in yielding meaningful answers to these questions. It will also become clear to what extent the blended intervention suits patients in specialised mental healthcare, by looking at adherence, dropout and satisfaction with treatment.

Another strength is the innovative character: the blended treatment in our study also includes a mobile component, making ecological momentary assessment and intervention techniques possible. The real-time monitoring of patient’s state can enhance insight into meaningful patterns of the depression, aspects contributing to changes in mood and influence of treatment interventions, acting as an intervention itself. With EMI, treatment engagement and completion of therapy-related homework tasks can also actively be encouraged, for example by sending reminders for scheduled activities. Adding these new possibilities, can aid in personalizing treatment and optimizing adherence [27, 75].

A further strong feature of this trial is that patients with comorbidity will only be excluded when alternative treatment for that comorbid condition is required primary to the treatment of MDD, similar to usual procedures in the treatment indication for depression. This will maximize the external validity of the trial and reflect the heterogeneity and complexity of the patient population in specialised routine mental healthcare settings. Also, we will not interfere with current practice within the TAU group. By following the usual care paths for depression, generalizability to routine practice is strengthened. This strength can also be regarded as a limitation, as TAU cannot be foreknown and is expected to be heterogeneous. However, this study is designed to be a pragmatic trial and it is our aim to compare the effectiveness of bCBT with current usual care. TAU will be monitored carefully, to control for potential confounding effects.

The current study also presents us the challenge of providing a new treatment modality within everyday practice. To aid the implementation and ensure treatment fidelity, therapists receive extensive training on how to deliver bCBT, (technical) support is offered by the research team and therapists are provided with a treatment manual. Taking into account the heterogeneity of the disorder, our blended protocol allows for some flexibility, e.g. in the order of the modules and time spent on each module. By keeping track of activities on the Moodbuster platform, we will be able to get insight into what happens during therapy to increase knowledge on the suitability and applicability of blended depression treatment for patients in specialised clinical practice.

Overall, this study will provide mental healthcare stakeholders evidence-based information on the clinical and cost-effectiveness of bCBT and recommendations on how bCBT can be integrated into routine specialised mental healthcare settings.

### Trial status

The trial is in the on-going recruitment phase. Recruitment started in July 2015.

### Ethics

The protocol for this study has been approved by the Medical Ethics Committee of the VU University Medical Centre (registration number 2015.078). The study is being conducted in Compliance with the Declaration of Helsinki [76]. Informed signed consent to participate in the study will be obtained from all participants.

### Consent for publication

Not applicable.

### Availability of data and materials

Not applicable.

## Abbreviations

bCBT: blended cognitive behavioural therapy
CBT: cognitive behavioural therapy
CEA: cost-effectiveness analysis
CEQ: Credibility and Expectancy Questionnaire
CONSORT: Consolidated Standards of Reporting Trials
CSQ: Client Satisfaction Questionnaire
CUA: cost-utility analysis
DSM: Diagnostic and Statistical Manual of Mental Disorders
EMA: ecological momentary assessment
EMI: ecological momentary intervention
FMI: fraction of missing information
FTF: face-to-face
iCBT: internet-based cognitive behavioural therapy
ICER: incremental cost-effectiveness ratio
INEP: Inventory of Negative Effects of Psychotherapy
IPT: interpersonal psychotherapy
ITT: intention to treat
M.I.N.I.: MINI International Neuropsychiatric Interview
MDD: Major Depressive Disorder
MICE: multiple imputation using chained equations
NTR: Nederlands Trial Register
PHQ: Patient Health Questionnaire
PP: per protocol
PTSD: post-traumatic stress disorder
QALYs: quality adjusted life years
QIDS-SR: Quick Inventory of Depressive Symptomatology Self-Report
RCT: randomised controlled trial
SPSS: Statistical Package for the Social Sciences
SUS: System Usability Scale
TAU: treatment as usual
TiC-P: Trimbos and iMTA Questionnaires on Costs Associated with Psychiatric Illness
WAI: Working Alliance Inventory
WHO: World Health Organisation

## References

[1] Lépine J-P, Briley M (2011). The increasing burden of depression. Neuropsychiatr Dis Treat.

[2] de Graaf R, Ten Have M, van Gool C, van Dorsselaer S (2011). Prevalence of mental disorders, and trends from 1996 to 2009. Results from NEMESIS-2. Tijdschr Psychiatr.

[3] Mathers CD, Loncar D (2006). Projections of global mortality and burden of disease from 2002 to 2030. PLoS Med.

[4] Patel V, Maj M, Flisher AJ, De Silva MJ, Koschorke M, Prince M (2010). Reducing the treatment gap for mental disorders: a WPA survey. World Psychiatry.

[5] Bijl RV, Ravelli A (2000). Psychiatric morbidity, service use, and need for care in the general population: results of the Netherlands Mental Health Survey and Incidence Study. Am J Public Health.

[6] Arnberg FK, Linton SJ, Hultcrantz M, Heintz E, Jonsson U (2014). Internet-delivered psychological treatments for mood and anxiety disorders: a systematic review of their efficacy, safety, and cost-effectiveness. PLoS One.

[7] Richards D, Richardson T (2012). Computer-based psychological treatments for depression: a systematic review and meta-analysis. Clin Psychol Rev.

[8] Spek V, Cuijpers P, Nyklícek I, Riper H, Keyzer J, Pop V (2007). Internet-based cognitive behaviour therapy for symptoms of depression and anxiety: a meta-analysis. Psychol Med.

[9] Gerhards SAH, de Graaf LE, Jacobs LE, Severens JL, Huibers MJH, Arntz A (2010). Economic evaluation of online computerised cognitive-behavioural therapy without support for depression in primary care: randomised trial. Br J Psychiatry.

[10] Johansson R, Andersson G (2012). Internet-based psychological treatments for depression. Expert Rev Neurother.

[11] Kleiboer A, Donker T, Seekles W, van Straten A, Riper H, Cuijpers P (2015). A randomized controlled trial on the role of support in Internet-based problem solving therapy for depression and anxiety. Behav Res Ther.

[12] Andersson G, Cuijpers P, Carlbring P, Riper H, Hedman E (2014). Guided Internet-based vs. face-to-face cognitive behavior therapy for psychiatric and somatic disorders: a systematic review and meta-analysis. World Psychiatry.

[13] Wagner B, Horn AB, Maercker A (2013). Internet-based versus face-to-face cognitive-behavioral intervention for depression: a randomized controlled non-inferiority trial. J Affect Disord.

[14] Andersson G, Hesser H, Veilord A, Svedling L, Andersson F, Sleman O (2013). Randomised controlled non-inferiority trial with 3-year follow-up of internet-delivered versus face-to-face group cognitive behavioural therapy for depression. J Affect Disord.

[15] Ruwaard J, Lange A, Schrieken B, Dolan CV, Emmelkamp P (2012). The effectiveness of online cognitive behavioral treatment in routine clinical practice. PLoS One.

[16] Williams AD, Andrews G (2013). The Effectiveness of Internet Cognitive Behavioural Therapy (iCBT) for Depression in Primary Care: A Quality Assurance Study. PLoS One.

[17] Andersson G, Hedman E (2013). Effectiveness of Guided Internet-Based Cognitive Behavior Therapy in Regular Clinical Settings. Verhaltenstherapie.

[18] Andrews G, Cuijpers P, Craske MG, McEvoy P, Titov N (2010). Computer Therapy for the Anxiety and Depressive Disorders Is Effective, Acceptable and Practical Health Care: A Meta-Analysis. PLoS One.

[19] Hadjistavropoulos HD, Pugh NE, Nugent MM, Hesser H, Andersson G, Ivanov M (2014). Therapist-assisted Internet-delivered cognitive behavior therapy for depression and anxiety: Translating evidence into clinical practice. J Anxiety Disord.

[20] Hedman E, Ljótsson B, Kaldo V, Hesser H, El Alaoui S, Kraepelien M (2014). Effectiveness of Internet-based cognitive behaviour therapy for depression in routine psychiatric care. J Affect Disord.

[21] Renton T, Tang H, Ennis N, Cusimano MD, Bhalerao S, Schweizer TA (2014). Web-Based Intervention Programs for Depression: A Scoping Review and Evaluation. J Med Internet Res.

[22] Beattie A, Shaw A, Kaur S, Kessler D (2009). Primary-care patients’ expectations and experiences of online cognitive behavioural therapy for depression: a qualitative study. Health Expect.

[23] Riper H, van Ballegooijen W, Kooistra L, de Wit J, Donker T (2013). Preventie & eMental-Health. Onderzoek dat leidt, technologie die verleidt, preventie die bereikt en beklijft.

[24] Kooistra LC, Wiersma JE, Ruwaard J, van Oppen P, Smit F, Lokkerbol J (2014). Blended vs. face-to-face cognitive behavioural treatment for major depression in specialized mental health care: study protocol of a randomized controlled cost-effectiveness trial. BMC Psychiatry.

[25] van der Vaart R, Witting M, Riper H, Kooistra L, Bohlmeijer ET, van Gemert-Pijnen L (2014). Blending online therapy into regular face-to-face therapy for depression: content, ratio and preconditions according to patients and therapists using a Delphi study. BMC Psychiatry.

[26] Wilhelmsen M, Lillevoll K, Risør MB, Høifødt R, Johansen ML, Waterloo K (2013). Motivation to persist with internet-based cognitive behavioural treatment using blended care: a qualitative study. BMC Psychiatry.

[27] Heron KE, Smyth JM (2011). Ecological Momentary Interventions: Incorporating Mobile Technology Into Psychosocial and Health Behavior Treatments. Br J Heal Psychol.

[28] Kramer I, Imons CLJPS, Artmann JEAH, Othmann CLME, Iechtbauer WOV, Eeters FRP (2014). A therapeutic application of the experience sampling method in the treatment of depression : a randomized controlled trial. World Psychiatry.

[29] Warmerdam L, Riper H, Klein M, van den Ven P, Rocha A, Ricardo Henriques M (2012). Innovative ICT solutions to improve treatment outcomes for depression: the ICT4Depression project. Stud Health Technol Inform.

[30] Andrade LH, Alonso J, Mneimneh Z, Wells JE, Al-Hamzawi A, Borges G (2014). Barriers to mental health treatment: results from the WHO World Mental Health surveys. Psychol Med.

[31] Andersson G, Cuijpers P (2008). Pros and cons of online cognitive-behavioural therapy. Br J Psychiatry.

[32] Høifødt R, Lillevoll K, Griffiths K, Wilsgaard T, Eisemann M, Waterloo K, Kolstrup N (2013). The Clinical Effectiveness of Web-Based Cognitive Behavioral Therapy With Face-to-Face Therapist Support for Depressed Primary Care Patients: Randomized Controlled Trial. J Med Internet Res.

[33] Månsson KNT, Skagius Ruiz E, Gervind E, Dahlin M, Andersson G (2013). Development and initial evaluation of an internet-Based Support System for Face-to-Face Cognitive Behavior Therapy: A Proof of Concept Study. J Med Internet Res.

[34] Ly KH, Topooco N, Cederlund H, Wallin A, Bergström J, Molander O (2015). Smartphone-Supported versus Full Behavioural Activation for Depression: A Randomised Controlled Trial. PLoS One.

[35] Kleiboer A, Smit J, Bosmans J, Ruwaard J, Anderson G, Topooco N, et al. European comparative effectiveness research on blended depression treatment versus treatment-as-usual (E-COMPARED): study protocol of a randomized controlled non-inferiority trial in eight European countries. In Press. 10.1186/s13063-016-1511-1 PMC4972947 27488181

[36] European Comparative Effectiveness Research on Internet-based Depression Treatment. 2015. http://www.e-compared.eu. Accesses 6 Jan 2016.

[37] Sheehan DV, Lecrubier Y, Sheehan KH, Amorim P, Janavs J, Weiller E (1998). The Mini-International Neuropsychiatric Interview (MINI): the development and validation of a structured diagnostic psychiatric interview for DSM-IV and ICD-10. J Clin Psychiatry.

[38] Kroenke K, Spitzer RL (2002). The PHQ-9 : A New Depression Measure. Psychiatr Ann.

[39] RANDOM.ORG (Randomness and Integrety Services Ltd) [IE]. 1998-2016. http://www.random.org. Accessed 28 Feb 2015.

[40] Moher D, Hopewell S, Schulz KF, Montori V, Gøtzsche PC, Devereaux PJ (2010). CONSORT 2010 Explanation and Elaboration: updated guidelines for reporting parallel group randomised trials. J Clin Epidemiol.

[41] Bockting CLH, Huibers MJH, Keijsers GJP, van Minnen A, Hoogduin CAL (2011). Protocollaire behandeling van patiënten met een depressieve stoornis. Protocollaire behandelingen voor volwassenen met psychische klachten 1.

[42] Spijker J, Bockting CLH, Meeuwissen JA, van Vliet IM, Emmelkamp PMG, Hermens MLM (2013). Multidisciplinaire richtlijn Depressie (Derde revisie). Richtlijn voor de diagnostiek, behandeling en begeleiding van volwassen patiënten met een depressieve stoornis.

[43] National Collaborating Centre for Mental Health (2010). Depression: the treatment and management of depression in adults (updated edition).

[44] Moodbuster. https://moodbuster.eu. Accessed 13 Jan 2016.

[45] ICT4Depression Deliverable 4.7: final evaluation report. 2013. http://www.ict4depression.eu/wp/wp-content/uploads/2011/03/FP7-248778-D4.7r1.pdf. Accesses 20 Feb 2016.

[46] Kroenke K, Spitzer RL, Williams JBW (2001). The PHQ-9. J Gen Intern Med.

[47] Wittkampf K, Naeije L, Schene AH, Huyser J, van Weert HC (2007). Diagnostic accuracy of the mood module of the Patient Health Questionnaire: a systematic review. Gen Hosp Psychiatry.

[48] Gilbody S, Richards D, Brealey S, Hewitt C (2007). Screening for depression in medical settings with the Patient Health Questionnaire (PHQ): A diagnostic meta-analysis. J Gen Intern Med.

[49] Rush AJ, Trivedi MH, Ibrahim HM, Carmody TJ, Arnow B, Klein DN (2003). The 16-Item Quick Inventory of Depressive Symptomatology (QIDS), clinician rating (QIDS-C), and self-report (QIDS-SR): a psychometric evaluation in patients with chronic major depression. Biol Psychiatry.

[50] Lecrubier Y, Sheehan DV, Weiller E, Amorim P, Bonora I, Sheehan KH (1997). The Mini International Neuropsychiatric Interview (MINI). A short diagnostic structured interview: reliability and validity according to the CIDI. Eur Psychiatry.

[51] Devilly GJ, Borkovec TD (2000). Psychometric properties of the credibility/expectancy questionnaire. J Behav Ther Exp Psychiatry.

[52] Pearlin LI, Schooler C (1978). The structure of coping. J Health Soc Behav.

[53] Deeg DJH, Huisman M (2010). Cohort differences in 3-year adaptation to health problems among Dutch middle-aged, 1992-1995 and 2002-2005. Eur J Ageing.

[54] Larsen DL, Attkisson CC, Hargreaves WA, Nguyen TD (1979). Assessment of client/patient satisfaction: development of a general scale. Eval Program Plann.

[55] Attkisson CC, Greenfield TK (1996). The client satisfaction questionnaire (CSQ) scales and the service satisfaction scale-30 (SSS-30). Outcomes assessment in clinical practice.

[56] Brooke J (1996). SUS - a quick and dirty usability scale. Usability Eval Ind.

[57] Bangor A, Kortum PT, Miller JT (2008). An Empirical Evaluation of the System Usability Scale. Int J Hum Comput Interact.

[58] Hatcher RL, Gillaspy JA (2006). Development and validation of a revised short version of the working alliance inventory. Psychother Res.

[59] Stinckens N, Ulburghs A, Claes L (2009). De werkalliantie als sleutelelement in het therapiegebeuren. Meting met behulp van de WAV-12: de Nederlandse vertaling van de Working Alliance Inventory. Tijdschr Klin Psychol.

[60] Ladwig I, Rief W, Nestoriuc Y (2014). What are the risks and side effects of psychotherapy? - development of an inventory for the assessment of negative effects of psychotherapy (INEP). Verhaltenstherapie.

[61] The EuroQol Group (1990). EuroQol-a new facility for the measurement of health-related quality of life. Health Policy.

[62] Herdman M, Gudex C, Lloyd A, Janssen M, Kind P, Parkin D (2011). Development and preliminary testing of the new five-level version of EQ-5D (EQ-5D-5L). Qual Life Res.

[63] Keeley T, Al-Janabi H, Lorgelly P, Coast J (2013). A Qualitative Assessment of the Content Validity of the ICECAP-A and EQ-5D-5L and Their Appropriateness for Use in Health Research. PLoS One.

[64] Janssen MF, Pickard AS, Golicki D, Gudex C, Niewada M, Scalone L (2013). Measurement properties of the EQ-5D-5L compared to the EQ-5D-3L across eight patient groups: a multi-country study. Qual Life Res.

[65] Hakkaart-van Roijen L, van Straten A, Tiemens B, Donker M (2002). Manual Trimbos/iMTA Questionnaire for Costs Associated with Psychiatric Illness (TiC-P).

[66] Cuijpers P, Turner EH, Koole SL, van Dijke A, Smit F (2014). What is the threshold for a clinically relevant effect? The case of Major Depressive Disorders. Depress Anxiety.

[67] Hedman E, Andersson G, Ljótsson B, Andersson E, Rück C, Mörtberg E (2011). Internet-based cognitive behavior therapy vs cognitive behavioral group therapy for social anxiety disorder: a randomized controlled non-inferiority trial. PLoS One.

[68] Schumi J, Wittes JT (2011). Through the looking glass : understanding non-inferiority. Trials.

[69] D’Agostino RB, Massaro JM, Sullivan LM (2002). Non-inferiority trials: design concepts and issues - the encounters of academic consultants in statistics. Stat Med.

[70] White IR, Royston P, Wood AM (2011). Multiple imputation using chained equations: Issues and guidance for practice. Stat Med.

[71] Rubin D (1987). Multiple imputation for nonresponse in surveys.

[72] Cohen J (1988). Statistical power analysis for the behavioral sciences.

[73] Smit F, Cuijpers P, Oostenbrink J, Batelaan N, de Graaf R, Beekman A (2006). Costs of nine common mental disorders: implications for curative and preventive psychiatry. J Ment Health Policy Econ.

[74] De Graaf R, Tuithof M, Van Dorsselaer S, Ten Have M (2012). Comparing the effects on work performance of mental and physical disorders. Soc Psychiatry Psychiatr Epidemiol.

[75] Wichers M, Simons CJP, Kramer IMA, Hartmann JA, Lothmann C, Myin-Germeys I (2011). Momentary assessment technology as a tool to help patients with depression help themselves. Acta Psychiatr Scand.

[76] World Medical Association (2013). World Medical Association Declaration of Helsinki: ethical principles for medical research involving human subjects. JAMA.

